# Preventing self-fertilization: Insights from *Ziziphus* species

**DOI:** 10.3389/fpls.2023.1226502

**Published:** 2023-08-18

**Authors:** Noemi Tel-Zur

**Affiliations:** French Associates Institute for Agriculture and Biotechnology of Drylands, Blaustein Institutes for Desert Research, Ben-Gurion University of the Negev, Beer Sheva, Israel

**Keywords:** dioecism, pre-or post-pollination mechanisms, self-incompatibility, synchronous dichogamy, hermaphroditism

## Abstract

The fitness of self-progeny individuals is inferior to that of their outcrossed counterparts, resulting in a reduction in a plant population’s ability to survive and reproduce. To prevent self‐fertilization, angiosperms with hermaphrodite flowers may exploit a variety of mechanisms, including synchronous dichogamy and self-incompatibility. Synchronous dichogamy involves two flowering morphs, with strict within-morph synchronization, thereby preventing not only autogamy and geitonogamy but also intra-morph mating. Self-fertilization is also prevented by self-incompatibility, a genetic mechanism that allows the identification and rejection of “self” pollen, thereby preventing both autogamy and geitonogamy. Here, I seek to provide a perspective of flowering in *Ziziphus* species exhibiting both synchronous (i.e., “Early” morph flowers open in the morning and “Late” morph flowers open in the afternoon) protandrous dichogamy (i.e., pollen dispersal before the stigma becomes receptive) and self-incompatibility.

## Introduction

Most angiosperm species produce hermaphrodite flowers, with both male and female organs in the same flower (Ainsworth, 2000; Renner, 2014). Hermaphroditism appears to increase pollination efficiency and fruit and seed set *via* self-pollination, i.e., transfer of pollen grains from the anther of one flower to the stigma of the same flower (autogamy) or to a genetically similar flower (geitonogamy), thus ensuring seed production. However, self-progeny plants exhibit reduced fitness, i.e., lower levels of heterozygosity, than their outcrossed counterparts, giving rise to a phenomenon known as inbreeding depression or a reduction in a population’s ability to survive and reproduce (Barrett, 1998; Kelly, 2005). To prevent self-pollination and hence self‐fertilization, plant species with hermaphrodite flowers often develop morphological, molecular and/or phenological adaptations (Lloyd and Webb, 1986; Bertin, 1993; Barrett, 1998; Opedal, 2018) that are exploited in pre- or post-pollination mechanisms [see Narbona et al., 2011 and references within).

The pre-pollination mechanisms are herkogamy and dichogamy. These two mechanisms are similar in that they both facilitate a separation of the presentation of mature anthers and stigmas and hence prevent self-fertilization (Lloyd and Webb, 1986; Wang H, et al., 2021). In herkogamy, morphological barriers provide a *spatial* separation of sexual functions, which reduces the possibility of intra-flower self-pollination; for example, hermaphrodite flowers may have long stamens and short styles or short stamens and long styles (Webb and Lloyd, 1986; Opedal, 2018). In contrast, in dichogamy, the maturation sequence of the sex organs in hermaphrodite flowers (Stout, 1928 and references within) results in a *temporal* separation of male and female functions. As such, there is no overlap between staminate and pistillate maturity in any particular flower, and that flower will thus be *functionally* male or female at a specific developmental time. Dichogamy, being a phenological adaptation that facilitates a temporal separation of male and female reproductive functions within each flower, is thus also referred to as “temporal dioecism” (Cruden, 1988; Molano-Flores, 2001). The term “protandrous dichogamy” is used when the male organs mature first, and “protogynous dichogamy,” when the first phase is female. Protandry is considered less effective in preventing self-fertilization, because pollen may often remain in the anthers and allow self-fertilization when stigmas become receptive (Bertin, 1993).

Dichogamy can be expressed at the whole plant level. As such, flower maturation and anthesis are synchronized at the whole plant level (synchronous dichogamy), meaning that pollen grains are released or stigmas mature within a small window of time (a few hours) in all the flowers on the plant (Narbona et al., 2011; Endress, 2020), producing two morphs with a reciprocal timing of male and female sexual functions. In other words, this developmental synchronization results in a “female” or “male” plant phase at a specific time of the day.

The post-pollination mechanism for preventing self-fertilization is the molecular mechanism of self-incompatibility, which is a genetic mechanism that is controlled by one or more multi-allelic loci and that relies on a series of cellular interactions to prevent self-fertilization (Allen and Hiscock, 2008; Fujii and Kubo, 2016). The self-incompatibility mechanism involves a process of self- and non-self-recognition between the pollen grain and the pistil, which will lead to inhibition of the fertilization of the self-pollen grain (Takayama and Isogai, 2005; Fujii and Kubo, 2016). Self-incompatibility enforces outcrossing, playing a vital role in species diversity in flowering plants (Goldberg et al., 2010).

While each one of the above-described adaptations is in itself thought to effectively prevent self-fertilization, some species have more than one mechanism, as is manifested in the model for this perspective—the hermaphroditic self-incompatible protandrous dichogamous flowers of *Ziziphus*. Since in protandrous flowers the possibility of self-fertilization by geitonogamy is higher than in protogynous, self-incompatibility may have evolved in response to geitonogamy (Porcher and Lande, 2005).

Here, I review the current literature addressing the role of synchronous dichogamy and self-incompatibility in preventing self-fertilization in *Ziziphus* species.

## Preventing selfing—*Ziziphus* as a case study

**Figure 1:**
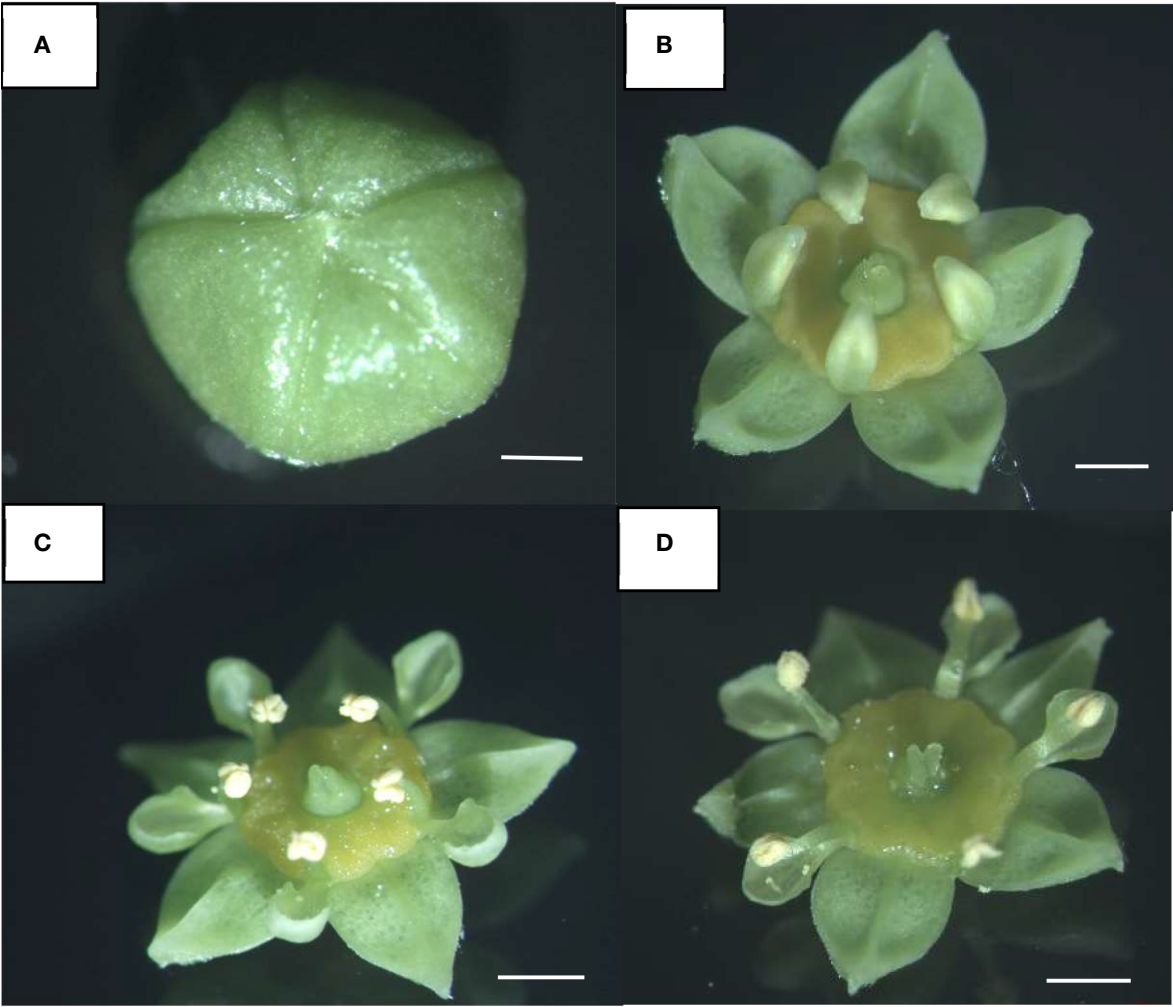
Floral developmental stages in *Ziziphus jujuba*. **(A)** Flower bud before opening. **(B)** Flower opening. **(C)** Male phase: erect stamens, open anthers, and short style. **(D)** Female phase: elongated pistil and developed stigma. Stamens recurve between the sepals. The developmental stages, i.e., anthesis and flower maturation, are synchronized at the whole plant level. Scale bar: 1 mm.

The diversity of mechanisms allowing/preventing sexual reproduction (reproductive strategies) in the short-lived (two-day) flowers of species of *Ziziphu*s (Rhamnaceae) (**Figure 1**) is well illustrated in studies of the morphology, physiology, genetics and reproduction of *Ziziphu*s (Galil and Zeroni, 1967; Lyrene, 1983; Weekley et al., 2002; Tel-Zur and Schneider, 2009; Asatryan and Tel-Zur, 2014; Cerino et al., 2015). Protandrous synchronous dichogamy was first reported in the evergreen species, *Z. spina-christi* (L.) Willd., commonly known as Christ’s thorn (Galil and Zeroni, 1967). Later, it was also reported in *Z. jujuba, Z. mucronata* and *Z. mauritiana*, all Old World *Ziziphus* species (Islam and Simmons, 2006). In these species, protandrous dichogamy is synchronized at the tree level, i.e., the flowers of each individual plant mature in synchronization, with little or no overlap between the sexual stages (Galil and Zeroni, 1967; Lyrene, 1983; Zietsman and Botha, 1992; Tel-Zur and Schneider, 2009; Wajnberg et al., 2019; Tel-Zur and Keasar, 2020). This rigid synchronization generates two genotype-specific morphs (**Table 1**)—one in which the male phase occurs in the morning (type A or “Early morph”) and the other in which the male phase occurs in the afternoon (type B or “Late morph”)—in a ratio of 1:1 in wild populations (Galil and Zeroni, 1967; Renner, 2001). The offspring of a hand pollination trial between the genotypes A and B segregated according to a ratio of 1:1 (“Early morph”: “Late morph”) (**Supplementary Data**), supporting the assumption that a single pair of alleles controls this trait (Renner, 2001 and references within).

**Table 1:** Synchronous dichogamy morphs. “Early” morph and “Late” morph in *Z. spina-christi* [in a ratio of 1:1 in wild populations, see Galil and Zeroni (1967)].

	Day 1			Day 2	
Morph A	Morning	Afternoon	Evening	Morning	Afternoon
	♂	♀	♀		
Morph B		♂	♀	♀	

In controlled hand self-pollination studies, a mechanism of self-incompatibility was identified in three *Ziziphus* species; in these trials, *Z. mauritiana* and *Z. spina-christi* produced fruits that dropped off soon after pollination or before maturation, while in *Z. jujuba* some flowers set small fruits lacking viable seeds (Galil and Zeroni, 1967; Lyrene, 1983; Asatryan and Tel-Zur, 2013; Wang F, et al., 2021). The presence of binucleate pollen grains and the cessation of pollen tube growth in the style observed in these three *Ziziphus* species support the assumption of a gametophytic self-incompatibility system (Asatryan and Tel-Zur, 2013). Self-incompatibility was also reported in *Z. mucronate* and *Z. celata* (Zietsman and Botha, 1992; Weekley and Race, 2001; Weekley et al., 2002; Cerino et al., 2015).

Two other *Ziziphus* species, both native to the New World, *Z. mistol* (Islam and Simmons, 2006) and *Z. celata*, also exhibit protandrous dichogamy, but the sexual phases overlap, i.e., there is a lack of synchronization at the plant level, thus potentially allowing geitonogamy and fertilization within a morph (Weekley et al., 2002; Cerino et al., 2015). In *Z. celata*, geitonogamy can occur, but self-fertilization is prevented by self-incompatibility (Weekley et al., 2002). In contrast, *Z. mistol* sets fruits and viable seeds after self-pollination, showing that this species is self-compatible, but a higher fruit set in obtained after cross-pollination (Cerino et al., 2015). Porcher and Lande (2005) developed a theoretical model to explain the breakdown of the gametophytic self-incompatibility system, showing that the spread of the self-compatible genotypes is favored by low or high selfing rates, a low number of *S*-alleles and pollen limitation. The fact that cross-pollination results in a higher fruit set in *Z. mistol* suggest a partial self-fertility, as reported in *Acca sellowiana* (Ramírez and Kallarackal, 2017). In *Leavenworthia alabamica* self-compatible plants were backcrossed into a self-incompatible population showing that self-compatible plants produced more seeds but those are less viable than outcrossed seeds, evoking that seed discounting and inbreeding depression may explain the fact that self-incompatibility are wide maintained also after selfing mutations in a population (Layman et al., 2017).

Synchronous dichogamy is a very effective mechanism to prevent self-fertilization, geitonogamy, and fertilization within a morph (Lyrene, 1983; Asatryan and Tel-Zur, 2013). However, a breakdown of the synchronization has been observed in natural populations of *Z. spina-christi* at the end of the flowering season (Galil and Zeroni, 1967), potentially allowing fertilization within a morph (but not via geitonogamy due to the self-incompatibility system) and thereby temporarily changing the species’ reproductive strategy to create a “window” for producing more seeds. To test this premise, Tel-Zur and Keasar (2020) investigated the outcome of hand cross pollination within trees of the same morph in a natural *Z. spina-christi* population. Fruit set was obtained, but at a significantly lower rate than open pollination. Important to note that the breakdown of the synchronization at the tree level was later reported in “Early morph” trees at the start of the flowering season, but it was particularly marked in both morphs at the end of the flowering season (Tel-Zur and Keasar, 2020), as was also reported by Galil and Zeroni (1967). A similar behavior was observed in the self-incompatible protandrous *Aconitum grossedentatum* (Ida and Minato, 2020). The collapse of the flower synchronization at the end of the flowering season suggests that synchronous protandry reduces only pre-pollination (selfing) events while post-pollination is blocked by the self-incompatibility system; showing that the benefit of the synchronous protandry decreases over the flowering season (Ida and Minato, 2020). Along with the breakdown of the flower synchronization in *Ziziphus* species, many of the flowers did not make the transition to the female phase due to the failure of the style to elongate and non-maturation of the stigma (Galil and Zeroni, 1967; Tel-Zur and Schneider, 2009; Tel-Zur and Keasar, 2020), resulting in a higher proportion of male-phase flowers. This phenomenon together with the breakdown of synchronization described above led to a marked alteration in the ovule:pollen grain ratio; which can contribute to the evolution of dioecy.

## Discussion

Since dichogamy has been interpreted as a mechanism for preventing inbreeding depression, the combination of dichogamy and self-incompatibility elicits questions about the functional significance of synchronous dichogamy. According to Bertin (1993), a study of angiosperm families revealed that the prevalence of dichogamy was similar in self-incompatible and self-compatible species, i.e., it was found in 73% of 239 self-incompatible species (from 59 families) and in 75% of 673 self-compatible species (from 89 families). Thus, Bertin postulated that – even if it can prevent self-fertilization – the main role of synchronous dichogamy does not lie in preventing self-fertilization but rather in other evolutionary mechanisms, such as preventing pollen-pistil interference or reducing pollen waste. It would thus appear that synchronous dichogamy provides only partial protection from self-fertilization (as evidenced by the breakdown of synchronization described above), whereas self-incompatibility provides complete protection. With this notion in mind, Routley et al. (2004) used published data to construct a phylogenetic framework. They did indeed show positive correlations between protandry (male function first) and self-incompatibility (as in *Ziziphus* species) and also between protogyny (female function first) and self-compatibility, leading to the conclusion that protandry probably evolved to reduce pollen-pistil interference, and protogyny, to reduce inbreeding. Therefore, it may be suggested that the principal function of synchronous dichogamy is to reduce anther-stigma interference, thus promoting efficient pollen dispersal (Lloyd and Webb, 1986; Harder et al., 2000). Notwithstanding this notion regarding the improved pollination efficiency in dichogamous species (which remains to be confirmed in future studies), it is clear that the temporal separation of floral sex morphs *per se* prevents, or strongly reduces, selfing. Indeed, the simultaneous occurrence of three mechanisms that prevent inbreeding, namely, dichogamy, synchronization of anthesis at the whole tree level, and self-incompatibility, seems to indicate complementary and redundant mechanisms to prevent self-pollination. For example, in a study of two *Euphorbia* species, geitonogamy was prevented in the self-compatible species *E. nicaeensis* due synchronized protogyny, but in *E. boetica* selfing was prevented due to partial self-incompatibility (Narbona et al., 2011). The authors of that study concluded that synchronous dichogamy and self-incompatibility cannot occur in the same species, since a single mechanism is sufficient to prevent (or strongly reduce) self-fertilization. However, the data collected in *Ziziphus* species stands in contradiction to this conclusion: in *Ziziphus* the functions of self-incompatibility and synchronous protandrous dichogamy do overlap (at least in part), although these redundant mechanisms relax at the breakdown of synchrony (Galil and Zeroni, 1967; Tel-Zur and Keasar, 2020), thus increasing the pool of potential reproductive partners for within-morph crossing. In keeping with this idea, trials using marker genes and floral manipulations have supported the assumption that herkogamy and dichogamy reduce self-pollination and promote pollen dispersal (Barrett et al., 2003).

The literature does not offer any clues to the evolutionary origin of the mechanisms that serve to prevent self-fertilization. However, the fact that some *Ziziphus* species have ‘abandoned’ synchronization and are self-compatible may be connected to the notion that synchronous dichogamy will probably lead to dioecy, i.e., to facilitating the evolution of separate sexes (Renner, 2001 and references within). In this regard, a pioneering study in *Z. spina-christi* used an evolutionary probabilistic model to test the possible role of insect pollinators in driving such an evolutionary process (Wajnberg et al., 2019). In that study, flower development patterns, floral food rewards, pollinator visits and fruit production were compared between “Early” and “Late” morphs. The data showed that the “Early” morph functions mainly as the pollen donor, while the “Late” morph sets more fruit (Wajnberg et al., 2019), suggesting that “Early” and “Late” morphs will specialize into male and female plants, respectively.

The molecular and genetic regulation of synchronous dichogamy are not yet understood. Further research is needed to uncover the specific genetic and molecular components that control synchronous dichogamy by studying the expression patterns of candidate genes, analyzing genetic mutants, and exploring the influence of environmental cues on reproductive success. The specific genes and molecular pathways involved in the timing and coordination of reproductive organ development remain to be elucidated. Further phylogenetic research aiming to understand the evolution and diversification of synchronous dichogamy would trace the evolutionary relationships among different plant species and identify patterns of trait evolution, including the presence or absence of self-incompatibility. In conclusion, synchronous protandrous dichogamy flowering may thus play a more complex role than previously appreciated in regulating the reproductive system in *Ziziphus* and other species.

## Data availability statement

The original contributions presented in the study are included in the article/**Supplementary Files**, further inquiries can be directed to the corresponding author.

## Author contributions

NT: Conceptualization, Writing.
