# Supplementary material for: Preventing self-fertilization: Insights from Ziziphus species
Source: Front Plant Sci. 2023 Aug 18;14:1226502. doi: 10.3389/fpls.2023.1226502 (PMC10471802; doi:10.3389/fpls.2023.1226502)
Supplement: Supplementary file 1 [file DataSheet_1.pdf]

SUPPLEMENTARY DATA

Supplementary Table S1: Morph segregation of the offspring following reciprocal hand cross-pollination between “Early” and “Late” morphs in *Ziziphus spina-christi.*

**Cross**<br>♀ **x** ♂	**“Early” morph**<br>**(%)**	**“Late” morph**<br>**(%)**
“Early” morph x “Late” morph	43	57
“Late” morph x “Early” morph	71	28
